# Tuning the Swelling Properties of Smart Multiresponsive Core-Shell Microgels by Copolymerization

**DOI:** 10.3390/polym11081269

**Published:** 2019-07-31

**Authors:** Timo Brändel, Maxim Dirksen, Thomas Hellweg

**Affiliations:** 1 Department of Physical and Biophysical Chemistry, Bielefeld University, Universitätstraße 25, 33615 Bielefeld, Germany; 2 Lund Institute of Advanced Neutron and X-ray Science (LINXS), IDEON Building: Delta 5, Scheelevägen 19, 22370 Lund, Sweden

**Keywords:** smart microgels, core-shell microgels, copolymerization, volume phase transition

## Abstract

The present study focuses on the development of multiresponsive core-shell microgels and the manipulation of their swelling properties by copolymerization of different acrylamides—especially *N*-isopropylacrylamide (NIPAM), *N*-isopropylmethacrylamide (NIPMAM), and NNPAM—and acrylic acid. We use atomic force microscopy for the dry-state characterization of the microgel particles and photon correlation spectroscopy to investigate the swelling behavior at neutral (pH 7) and acidic (pH 4) conditions. A transition between an interpenetrating network structure for microgels with a pure poly-*N*,*n*-propylacrylamide (PNNPAM) shell and a distinct core-shell morphology for microgels with a pure poly-*N*-isopropylmethacrylamide (PNIPMAM) shell is observable. The PNIPMAM molfraction of the shell also has an important influence on the particle rigidity because of the decreasing degree of interpenetration. Furthermore, the swelling behavior of the microgels is tunable by adjustment of the pH-value between a single-step volume phase transition and a linear swelling region at temperatures corresponding to the copolymer ratios of the shell. This flexibility makes the multiresponsive copolymer microgels interesting candidates for many applications, e.g., as membrane material with tunable permeability.

## 1. Introduction

In soft matter research, stimuli-responsive core-shell microgels have become a strongly investigated field in recent years [1,2,3]. Different particle architectures have been examined in this context, ranging from dual-responsive networks [4,5,6,7], amphiphilic self-assembled systems [8], colloidally supported nanoreactors [9,10], nanoparticle containers [11,12], interpenetrating networks [13,14], and hollow spheres [15,16] to core-shell microgels with natural materials (e.g., starch) [17]. Additionally, core-shell microgels with different charges inside the core and the shell have been presented [18,19].

One of the most prominent examples for stimuli-responsive microgels are thermoresponsive particles based on poly-*N*-isopropylacrylamide (PNIPAM). At a certain temperature, the so-called volume phase transition temperature (VPTT), PNIPAM-based microgels undergo a reversible change in size, which is connected to a microphase separation of the polymer network [20,21]. One of the most advantageous properties of PNIPAM based microgels is the possibility to tailor their stimuli-responsive behavior by copolymerization [22,23]. For instance, it is easily possible to add a pH-response to the microgel particles by adding acrylic acid (AAc) as a comonomer during the precipitation polymerization [24,25]. In this context, not only PNIPAM-based microgels are investigated, but also microgels composed of poly-*N*-isopropylmethacrylamide (PNIPMAM) and poly-*N*,*n*-propylacrylamide (PNNPAM) have recently been extensively examined [26,27,28,29]. Similar to linear chains of PNIPAM, which show a coil-to-globule transition, PNIPAM microgels exhibit a deswelling process when the LCST of the polymer is reached. However, this so-called volume phase transition (VPT) is rather continuous in most cases, most likely due to the distribution of the degree of polymerization of the chains inside the network [30].

The volume phase transition of thermoresponsive microgels is affected by different particle properties. Firstly, the chemical composition is an important parameter for the swelling behavior of the microgels. Wedel et al. [31] have shown that the VPTT of copolymer microgels composed of PNIPMAM and PNNPAM can be tailored between the VPTTs of the pure microgel particles by adjusting the molar ratios of the two monomers. For a comparable system based on PNIPAM and PNIPMAM, Keerl et al. [32] were able to describe the particle form-factor of these copolymer microgels at temperatures in between the volume phase transition with a so-called “dirty-snowball” form-factor. In both cases, a cooperative volume phase transition of the network was predicted, which was confirmed by temperature dependent FTIR measurements in solution [33].

Secondly, the particle architecture of the microgels is of great importance for the volume phase transition. A versatile approach to design core-shell particles was developed by Jones et al. [34]. The authors used a two-step precipitation polymerization, in which the premade core microgel were used as seed particles for the precipitation of the polymer shell. Usually, no secondary nucleation occurs in this process and it also nicely works for inorganic seed particles [11]. Therefore, it became a standard procedure for the synthesis of core-shell microgels—which was also used, for example, by Berndt et al. [35], to show that a two-step phase transition or a linear phase transition region can be realized in core-shell microgels composed of materials with different VPTTs; and by Zeiser et al. [36], who analyzed the influence of the cross-linker density of the core on the linear phase transition region. It is still under investigation, which structural properties cause the appearance of the linear phase transition region. Berndt et al. [37] showed that only a small region of interpenetration is formed during the polymerization if PNIPAM is used as shell material. On the other hand, Wiehemeier et al. [33] deduced from temperature dependent FTIR measurements, that the use of PNNPAM as shell monomer increases the formation of interpenetrated regions. In addition, the influence of various comonomers on core-shell and hollow core-shell structures has e.g., been investigated by Lapeyre et al. [38].

In recent work, using the well established core-shell synthesis procedure by Jones et al. [34], Berndt et al. [35] and Zeiser et al. [36], it was shown that it can be used to generate versatile carriers for catalytically active silver nanoparticles [39]. These core-shell microgels consisted of a PNIPAM–*co*–AAc core and a PNNPAM shell, and exhibited a very interesting swelling behavior with two different phase transition regions at neutral pH-values. Cryo-transmission electron microscopy measurements showed that the core-shell structure of the carrier molecules is preserved through the decoration of the carriers with silver nanoparticles. In compareable works, Suzuki et al. [40,41] found similar swelling behavior and structure for core-shell microgels with PNIPAM–*co*–acrylamide cores, with PNIPAM shells decorated with gold nanoparticles.

In this work, we present a straightforward concept to further modify the volume phase transition of the previously mentioned core-shell microgels by using a copolymer microgel-shell consisting of PNNPAM–*co*–PNIPMAM networks. The results are multiresponsive core-shell microgels with a PNIPAM–*co*–AAc@PNNPAM–*co*–PNIPMAM architecture. These core-shell microgels are analyzed with atomic force microscopy (AFM), and the swelling behavior of the microgel particles is investigated by means of photon correlation spectroscopy (PCS). Particular attention is paid to the influence of the pH-value on the volume phase transition of the multiresponsive core-shell microgels by performing PCS measurements at neutral (pH 7) and acidic (pH 4) conditions.

## 2. Results and Discussion

All core-shell microgels synthesized in this work comprise the same core material, consisting of a PNIPAM–*co*–AAc core with an nominal acrylic acid content of 5 mol % and a nominal cross-linker content of 10 mol %. The cross-linker content of the shell is also kept constant (7.5 mol %). The investigated core-shell microgels, the chemical composition of the shells, and their sample names are presented in Table 1. Exact synthesis protocols can be found in Section 4.2.

### 2.1. Dry-State Particle Characterization

**Figure 1:**
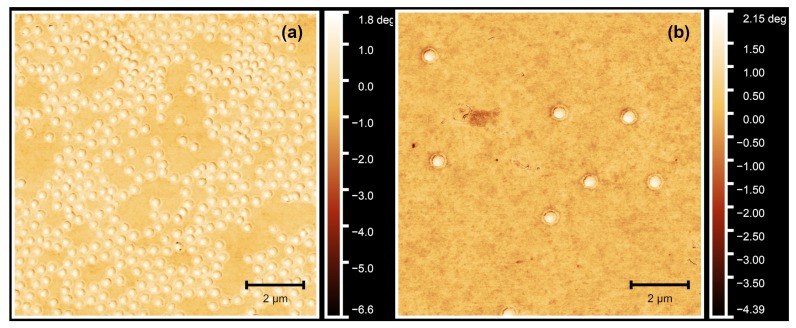
Atomic force microscopy (AFM) phase images of the PNIPAM–*co*–AAc core microgels (**a**) and, as a typical example, the core-shell microgel sample PNN50/PMAM50 (**b**). In (**b**), the corona formed in the second precipitation polymerization step can be clearly identified. All measurements were performed in the dry state.

**Figure A2:**
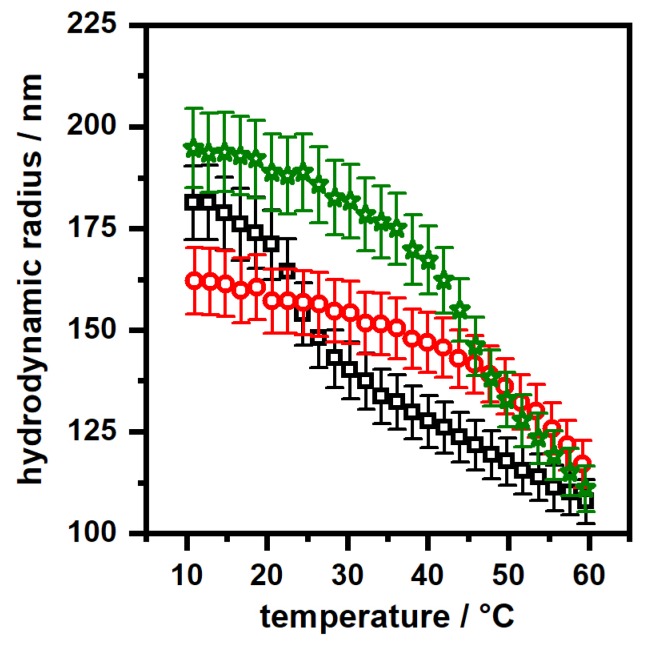
Comparison of the PCS swelling curves of the core microgels (red circles) and the core-shell particles PNN100/PMAM0 (black squares) and PNN0/PMAM100 (olive stars). All measurements were performed at pH7.

To verify the success of the core-shell microgel synthesis, we performed atomic force microscopy (AFM) on dried microgel samples on silicon surfaces. Due to the different cross-linker contents of core and shell, a difference between both should be observable in the phase contrast of the AFM images. Figure 1 displays a comparison between the phase images of the pure-core microgels and the core-shell particles. First of all, it is obvious that the particles in Figure 1b are bigger compared to the seed particles shown in Figure 1a. This arises from the formation of the shell and is in good agreement with observed growth of the hydrodynamic radii (see also Figure A2 in Appendix A). Second, only one particle species is observed.

Moreover, as expected, only one phase difference is observable in Figure 1a, which is the difference between the microgel particles and the silicon surface. On the outside of the microgel particles, one might recognize a very thin region of phase difference between the surface and the particle. This can be attributed to a small corona which is typical for PNIPAM–*co*–AAc microgels [42]. In contrast to that, an additional and very pronounced phase difference can be observed for the core-shell particles between the microgel core and the shell (Figure 1b). Comparable observations have often been described for core-shell microgel systems with inorganic core particles, e.g., silica cores [11]. In this case, the phase difference is less pronounced compared to a inorganic core, due to the polymeric nature of both core and shell. However, the microgel core and shell still exhibit a different interaction with the AFM probe due to their different cross-linker content. All in all, the synthesis of the core-shell particles was successful.

**Figure 2:**
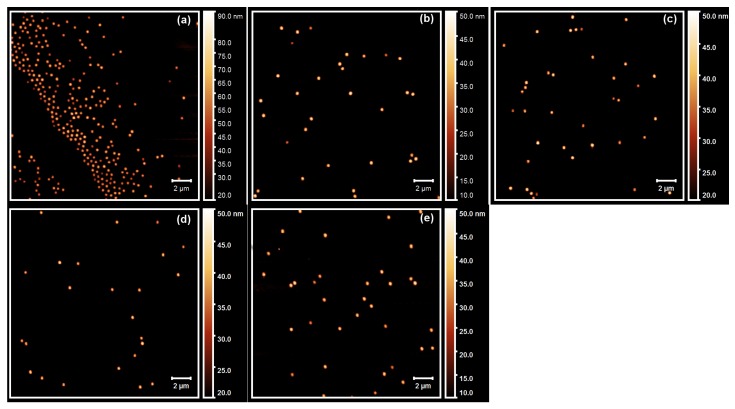
AFM images of all core-shell microgels with a PNIPAM-*co*-AAc core and a shell composed of (**a**) PNN100/PMAM0; (**b**) PNN75/PMAM25; (**c**) PNN50/PMAM50; (**d**) PNN25/PMAM75 and (**e**) PNN0/PMAM100.

To characterize all core-shell microgels in more detail, we also performed AFM height measurements of all samples on dried silicon surfaces. The resulting AFM height images are shown in Figure 2.

In all AFM height images displayed in Figure 2, particles with a circular cross section are observable. Therefore, a particle size distribution analysis is possible. We analyzed the particle size distribution for all samples with the free software package ImageJ [43]. The averaged lateral particle radii $R_{\mathrm{AFM}}$ are shown in Table 2.

All core-shell microgel samples seem to exhibit a standard deviation of the averaged lateral radius $R_{\mathrm{AFM}}$ between 23 and 32 nm, which seems to be rather high for a typical microgel sample. However, the polydispersity of the sample is still in an acceptable range, between 15% and 17%, for all core-shell microgels containing PNIPMAM in the shell. The highest standard deviation is found for the core-shell microgel with the pure PNNPAM shell. Nevertheless, no distinct second particle species is observable in case of the sample PNN100/PMAM0, and it seems possible that the apparent width of the size distribution could be caused by the low number of particles analyzed in the images. As a result of that, we performed a polydispersity index (PDI) analysis of the angle-dependent PCS measurements, which is presented in Section 2.2.

**Figure 3:**
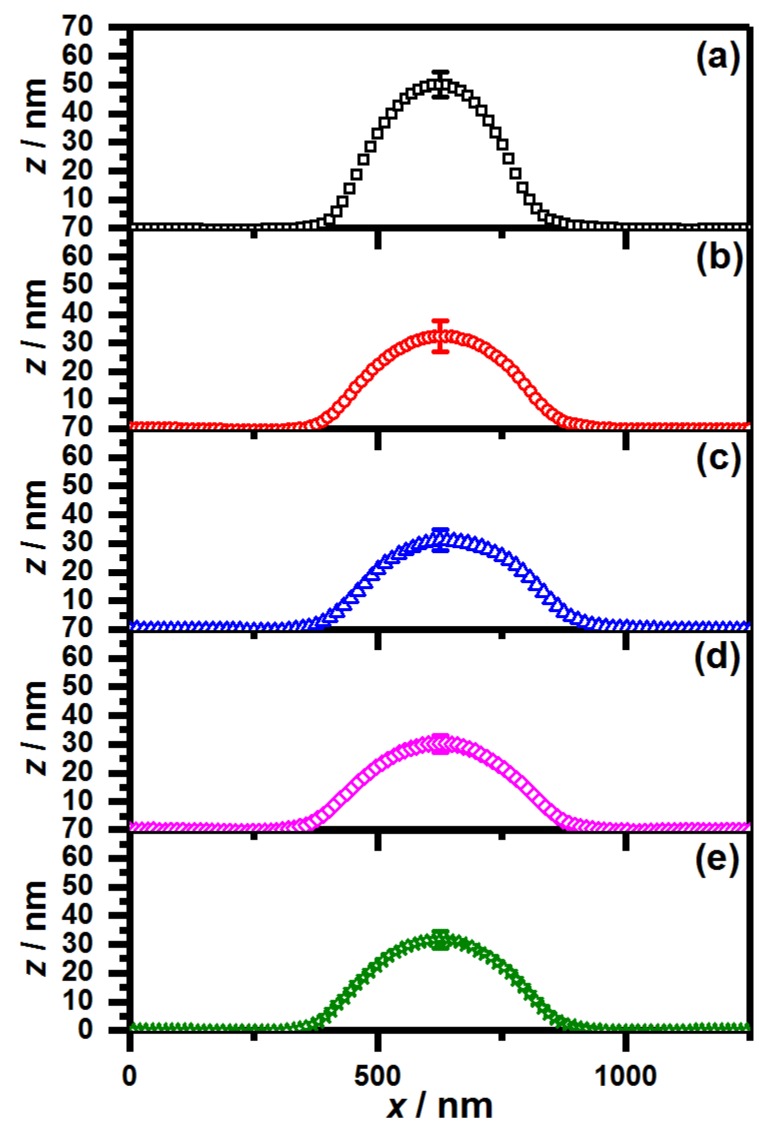
Averaged AFM height profiles of all core-shell microgels with a PNIPAM-*co*-AAc core and a shell composed of (**a**) PNN100/PMAM0; (**b**) PNN75/PMAM25; (**c**) PNN50/PMAM50; (**d**) PNN25/PMAM75 and (**e**) PNN0/PMAM100. The error bar was extracted from the standard deviation of all individual height profiles at the particle maximum.

Another interesting difference occurs between the samples, which contain PNIPMAM in the shell and the sample with the pure PNNPAM shell when the particle size is investigated. The sample PNN100/PMAM0 shows a significantly reduced lateral radius $R_{\mathrm{AFM}}$ compared to the other samples. It seems straightforward to conclude that the particle size of the respective sample is lower than the size of all other core-shell microgels. However, an important point, which has to be considered when analyzing the particle size on a surface, is the lateral extension of the microgels on the surface [44]. To investigate the extension of the particles on the surface, we extracted the height profiles of all core-shell particles from the AFM measurements. The results are presented in Figure 3.

Figure 3 shows that the height of all core-shell microgels containing PNIPMAM is significantly lower compared to the core-shell microgels with the pure PNNPAM shell. Hence, in contrast to the previous observations considering the lateral radii, it has to be concluded that all analyzed core-shell microgels have approximately the same size because the core-shell microgels with the lower lateral expansion exhibit an increased height in the AFM height profiles. Additionally, important information about the particle rigidity can be extracted from the inferior extension of the core-shell microgels with a pure PNNPAM shell. Obviously, the particles from the sample PNN100/PMAM0 are less deformable than the core-shell microgels containing PNIPMAM in the shell, although the initial cross-linker content of the core and the shell was kept constant in all cases. This can be concluded by the difference of the lateral radii and the height profiles of the individual microgels. While the difference between lateral radius and the maximum height is 115±9 nm for all microgels containing PNIPMAM in the shell, it is only 58 nm for the microgels with the pure PNNPAM shell.

A possible explanation of this could be an interpenetration of the shell polymer in the core region of the microgels. Wiehemeier et al. [33] showed, via temperature-dependent FTIR-measurements, that PNIPMAM@PNNPAM core-shell microgels exhibit an interpenetrated region in their architecture, which is not observed for the inverse PNNPAM@PNIPMAM system. In this case, the core material consists of PNIPAM–*co*–AAc and it has been shown that these microgels tend to form core-corona architectures [42,45], which would be beneficial for the formation of the mentioned interpenetrated regions. Following this, we suppose that the pure PNNPAM shell shows the strongest interpenetration and therefore leads to particles with a significantly increased rigidity, while the presence of PNIPMAM induces a less pronounced interpenetration resulting in softer particles.

However, all these observations are only made for particles in the dry state absorbed on a surface, and it is possible that the swelling of the particles with water influences the structure of the particles. Hence, we investigated the temperature- and pH-dependent swelling behavior of all core-shell microgels by photon correlation spectroscopy, which is presented in the following section.

### 2.2. Swelling Behavior

**Figure 4:**
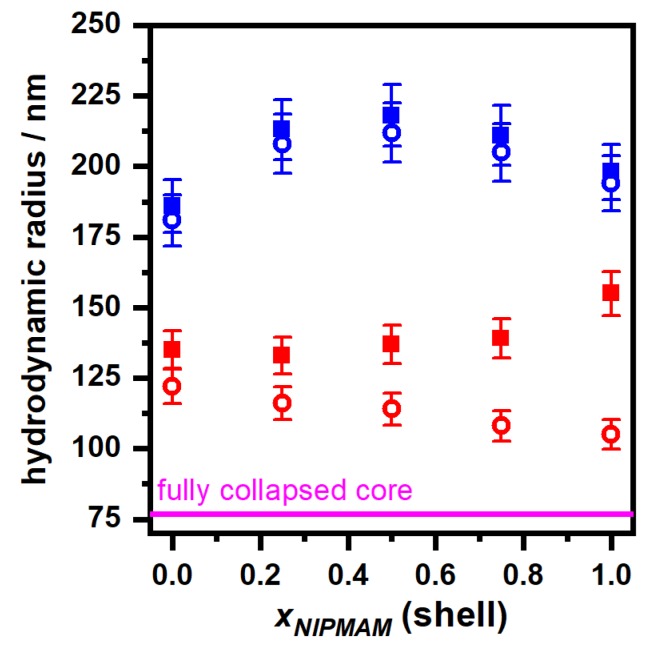
Plot of the hydrodynamic radii of all core-shell microgels in the fully swollen state (10 °C, blue symbols) and in the collapsed state (55 °C, red symbols) against the PNIPMAM fraction of the shell at pH = 7 (squares) and pH = 4 (circles). The magenta line indicates the hydrodynamic radius of the fully collapsed core (pH = 4, 55 °C). All hydrodynamic radii were obtained from angle-dependent PCS measurements.

The hydrodynamic properties of all core-shell microgels are investigated by performing angle-dependent photon correlation spectroscopy (PCS) measurements in the swollen (10 °C) and in the collapsed (55 °C) state at neutral (pH 7) and acidic (pH 4) conditions. All measurements led to linear relations between the relaxation rate Γ and $q^{2}$ with almost no deviations (see Appendix A). Consequently, we calculated the hydrodynamic radii of the core-shell microgels and plotted these as a function of the molar PNIPMAM fraction of the shell. The results are displayed and compared to the size of the fully collapsed core (pH = 4, 55 °C) in Figure 4.

The hydrodynamic radii of all core-shell microgels in the swollen state, displayed in Figure 4, show a comparable trend as the AFM measurements in the dry state. It seems that all microgels exhibit roughly the same hydrodynamic radius of about 190–220 nm. If the pH-value is lowered from 7 to 4, the hydrodynamic radius of all core-shell microgels decreases slightly, although the effect seems to be nearly negligible. This observation is in line with other measurements of core-shell microgels with acrylic acid in the core [34]. As mentioned before, we also performed an analysis of the polydispersity of all samples in the swollen state (see Table 3) to compare the results with the AFM image analysis.

In case of the PCS measurements we observe a very low polydispersity index (PDI), which is below 5% for all samples. This is a typical result for core-shell microgels synthesized via the two-step precipitation polymerization [37], but seems to be contradictory to the particle size analysis of the AFM images of the core-shell microgels. However, it has to be mentioned that only a rather low number of particles was analysed by AFM. Additionally, the particle size analysis of Image J relies heavily on the contrast between the particles and the surface, which could lead to errors when the particles spread on the surface during deposition as the core-shell microgels do. Hence, we think the results of the angle-dependent PCS measurements are more appropriate to compute a realistic PDI of the core-shell microgels.

If the angle-dependent PCS measurements are performed at 55 °C (red symbols in Figure 4), the influence of the copolymerization of PNNPAM and PNIPMAM on the properties of the core-shell microgels becomes clearly visible. To compare the rigidity of the particles with the results extracted from the AFM height profiles, we analysed the difference in the hydrodynamic radius of the particles in the fully swollen state (pH 7, 10 °C) and in the fully collapsed state (pH 4, 55 °C). As expected from the AFM results, the core-shell microgels with the pure PNNPAM shell exhibit the lowest difference in the hydrodynamic radii, compared to all particles with PNNPAM–*co*–PNIPMAM shells. Similar observations are possible, when the size of the fully collapsed core-shell particles is compared to the size of the fully collapsed core microgels. The size difference between the core-shell particles with the pure PNNPAM shell is larger, than for the system with the pure PNIPMAM shell. Consequently, the particles from the sample PNN100/PMAM0 have to be the most rigid core-shell samples investigated in this study.

A very useful parameter to describe the swelling of the core-shell microgels is the maximum swelling ratio $\alpha_{\max}$, which is calculated via Equation (1). In the present study we use this parameter to investigate the influence of the pH-value on the swelling behavior of all core-shell microgels.

(1) $$\alpha_{\max}=\frac{R_{h,10\;{}^{\circ}C}^{3}}{R_{h,55\;{}^{\circ}C}^{3}}$$

**Figure 5:**
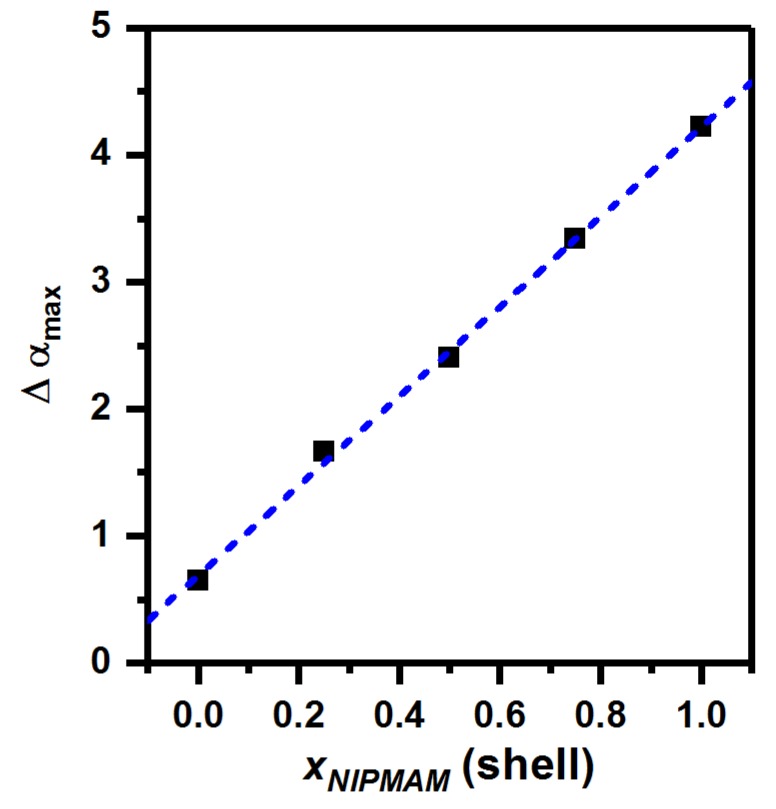
$\Delta \alpha_{\max}$ values, computed via Equations (1) and (2) plotted against the PNIPMAM fraction of the microgel-shell. The dashed blue line represents a linear fit of the data.

A comparison of the changes in the hydrodynamic radii of all core-shell particles at pH 7 and pH 4 leads to the very interesting observation, that the difference between the maximum swelling ratios seems to be influenced by the amount of PNIPMAM present in the microgel shell. The higher the molar ratio of PNIPMAM, the higher the difference between $\alpha_{\max,\mathrm{pH}=4}$ and $\alpha_{\max,\mathrm{pH}=7}$. To investigate the influence of the PNIPMAM ratio on the pH dependent swelling behavior in detail, we calculated the $\Delta \alpha_{\max}$-values (see Equation (2)) and plotted them against the molfraction of PNIPMAM in the shell (Figure 5).

(2) $$\Delta \alpha_{\max}=\alpha_{\max,\;\mathrm{pH}\;=\;4}-\alpha_{\max,\;\mathrm{pH}\;=\;7}$$

Figure 5 reveals a very interesting linear dependence of $\Delta \alpha_{\max}$ on the molar ratio of PNIPMAM in the shell. This means, that the influence of the pH-value on the swelling of the core-shell microgels is strongly influenced by the chemical composition of the shell. Precisely, the higher the amount of PNIPMAM in the shell, the higher the difference of the maximum swelling-ratios at pH 4 and at pH 7. We suppose that this phenomenon can be explained by the different tendencies to form layers of intepenetrating networks of core and shell, which were observed in AFM measurements. A strong interpenetration of the shell inside the core network reduces the charge density of the microgel network and increases the hydrophobicity of the interpenetrated regions. Due to the strong interpenetration of PNNPAM in the core-region of the microgels two effects are caused: Firstly the particle rigidity increases, which causes a reduced maximal swelling capacity. This is supported by the fact, that the hydrodynamic radius of all particles at pH4 in the collapsed state decreases with increasing PNIPMAM ratio. Secondly, the deswelling process of the shell seems to trigger the collapse of the core-network in the interpenetrated regions, even if the carboxylic moieties are deprotonated. Hence, the effect of the pH-value on the swelling capacity of the microgels is reduced by the interpenetration.

To verify our assumptions concerning the different states of interpenetration of the core-shell microgels, we calculated the radius of gyration $R_{g}$ from the average scattering intensity at low scattering angles by using the Guinier-approximation for the microgels with a pure PNNPAM shell and a pure PNIPMAM shell in the swollen state at pH 7. The respective values are 139 nm for the sample PNN100/PMAM0 and 134 nm for the sample PNN0/PMAM100. This leads to ratios of $\frac{R_{g}}{R_{h}}$ of 0.73 and 0.67, respectively. From this result it can be deduced that, in accordance with our suggestion, the core-shell microgels with a pure PNNPAM shell exhibit a more compact structure compared to the core-shell microgels with a pure PNIPMAM shell.

**Figure 6:**
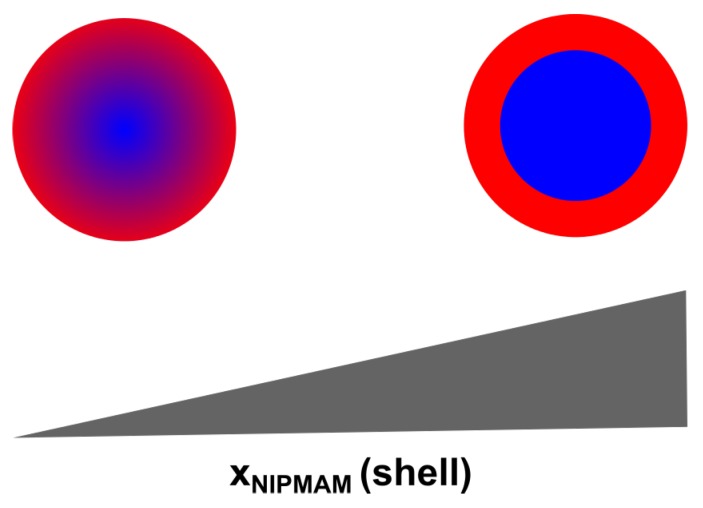
Schematic representation of the structural changes in the core-shell microgels as a function of the PNIPMAM fraction of the shell.

To sum up the results obtained from the angle-dependent measurements, it can be stated that we synthesized core-shell microgels with stimuli-responsive behavior concerning temperature and pH-value with a low polydispersity. We were able to show, that copolymerization of PNNPAM and PNIPMAM in the shell network influences the structure of the core shell microgels, in particular the interpenetrating layer of core and shell. The higher the PNIPMAM fraction of the shell, the more pronounced the core-shell structure of the microgels becomes as schematically displayed in Figure 6.

**Figure 7:**
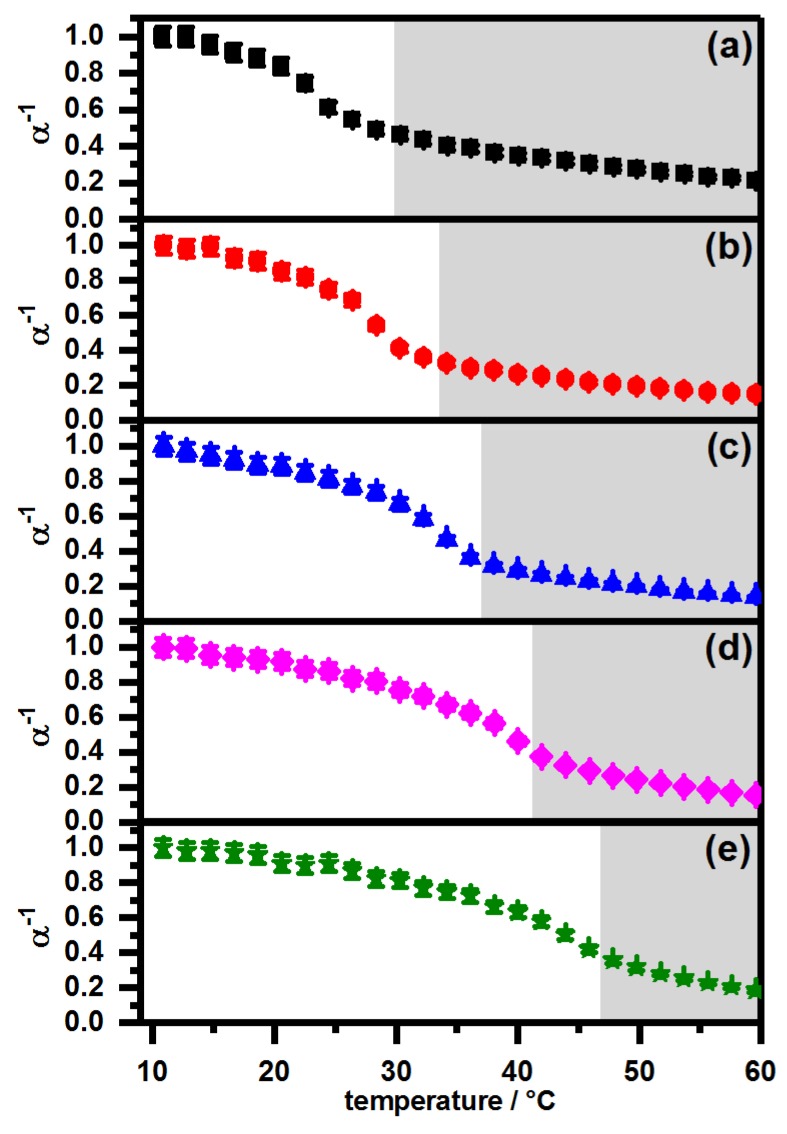
PCS swelling curves at pH 7 of the samples (**a**) PNN100/PMAM0 (black squares); (**b**) PNN75/PMAM25 (red circles); (**c**) PNN50/PMAM50 (blue triangles); (**d**) PNN25/PMAM75 (magenta diamonds) and (**e**) PNN0/PMAM100 (olive stars). The region of the linear volume phase transition is highlighted by a grey background.

In addition to the angle dependent PCS measurements we measured the swelling curves of all core-shell microgels at a constant scattering angle at pH 7 and pH 4, to investigate the swelling behavior of the particles in detail. The swelling curves of the core-shell microgels measured at pH 7 are shown in Figure 7. To improve comparability of all measurements, we plotted the $\alpha^{-1}$-values, calculated by $R_{h}^{3}/R_{h,10\;{}^{\circ}C}^{3}$, of all core-shell microgels against the temperature. A comparison between the swelling behavior of the core microgel and the core-shell systems PNN100/PMAM0 and PNN0/PMAM100 at neutral pH can be found in the Appendix A (see Figure A2).

The swelling curves in Figure 7 show that the deswelling process of all core-shell microgels is comparable at neutral pH-values. In all cases, the swelling curves exhibit three regions. At first, the particles are in the swollen state and do not show a remarkable deswelling. Afterwards, the main volume phase transition, which occurs at the LCST of the shell polymer, is observed. The temperature where this transition occurs is linearly dependent on the fractions of PNNPAM and PNIPMAM in the shell, which is in full agreement with the observations by Wedel et al. [31] for copolymer microgels. The third part of the swelling curves, displayed in Figure 7, is a linear phase transition region which was already observed before [39], and a plateau is not reached for any of the core-shell microgels. In this context, consequently the length of the linear phase transition region decreases with increasing PNIPMAM fraction of the shell, because the temperature of the main phase transition is shifted towards higher values. Moreover, it is very important to notice that no two-step phase transition is observable for any of the samples.

**Figure 8:**
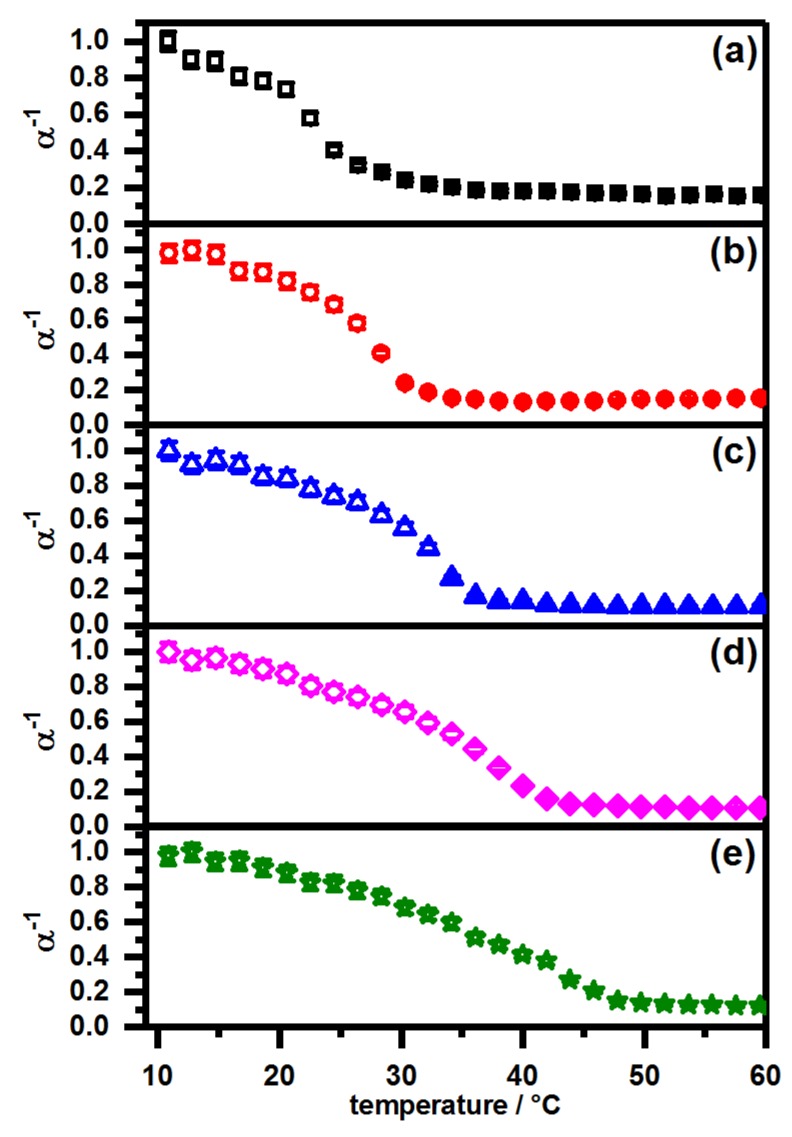
PCS swelling curves of the samples (**a**) PNN100/PMAM0 (black squares); (**b**) PNN75/PMAM25 (red circles); (**c**) PNN50/PMAM50 (blue triangles); (**d**) PNN25/PMAM75 (magenta diamonds) and (**e**) PNN0/PMAM100 (olive stars). All measurements were performed at pH4.

As we wanted to pay particular attention to the pH-dependence of the swelling behavior, we also measured the swelling curves of all core-shell microgels at pH 4. The results are displayed in Figure 8.

From the swelling curves in Figure 8, it is obvious that the swelling behavior of the core-shell microgels strongly depends on the pH-value. Usually, a two step volume phase transition would be observed for all systems which exhibit a microgel shell with a higher VPTT compared to the microgel core, as it was described by Berndt et al. [37], but in our case, only the core-shell microgel with a pure PNIPMAM shell seems to show this behavior. Instead, the particles tend to exhibit a rather strong, almost linear osmotic deswelling prior to the volume phase transition of the shell. Due to the acidic conditions, the broadening of the volume phase transition of the microgel core is suppressed due to the protonation of the carboxylic moieties and obviously the deswelling process of the core-shell system is now clearly dominated by the volume phase transition of the shell. Hence, the main volume phase transition occurs at temperatures which correspond to the VPTT of copolymer microgels of PNNPAM and PNIPMAM, as predicted by Wedel et al. [31]. In contrast to the swelling curves at pH 7 (see Figure 7), no linear decrease in size is observable for all core-shell microgels beyond the main volume phase transition in Figure 8.

The different observations compared to the core-shell microgels prepared by Berndt et al. are caused by the copolymerization of acrylic acid in the microgel core. As the synthesis of the shell is performed under neutral conditions in purified water, the carboxylic moieties in the seed particles are partially deprotonated. It is possible that the seed particles are not in the fully collapsed state even under the synthesis conditions at 80 °C. This obviously influences the resulting core-shell microgel structure and should be investigated in further studies, e.g., with small-angle neutron scattering and the analysis of the data with a reverse Monte-Carlo approach [46]. Another possibility could be the use of super-resolution fluorescence microscopy, which proved to be a powerful tool to investigate the network density of microgel particles [47,48]. Combining these techniques, a full understanding of the structural differences between the mentioned particles could be possible. Nevertheless, our assumption is supported by the fact that the core-shell microgels with the most distinct core-shell structure (pure PNIPMAM shell) seem to show the expected two-step volume phase transition.

As a brief summary, it can be stated that the swelling behavior of the core-shell particles can be manipulated by different strategies. First of all, it is possible to tune the VPTT of the main volume phase transition of all particles by copolymerization of PNNPAM and PNIPMAM in the microgel shell. Furthermore, it is possible to completely switch on and off the presence of a linear phase transition region after the main volume phase transition by varying the pH-value.

## 3. Conclusions

In this study, we present a copolymerization based approach to tune the swelling behavior of multiresponsive PNIPAM–*co*–AAc@PNNPAM–*co*–PNIPMAM core-shell microgels. It is shown that the particles with a pure PNNPAM shell are rather rigid, compared to the microgels with increasing molar ratios of PNIPMAM in the shell. This can be explained assuming an increased formation of an interpenetrating network structure in relation to the PNNPAM content. Furthermore, an investigation of the maximum swelling ratio with photon correlation spectroscopy at acidic (pH 4) and neutral (pH 7) conditions revealed a linear dependency of the $\Delta \alpha_{\max}$ values on the PNIPMAM content of the shell, which is also caused by the different grade of interpenetration in all core-shell microgels. A temperature dependent investigation of the swelling behavior of all core-shell microgels at pH 4 and pH 7 has shown that the swelling behavior can be shifted drastically from a single-step phase transition under acidic conditions to a two-phase volume phase transition consisting of a main phase transition followed by a broad linear phase transition region at neutral pH-values. In all cases, the VPTT of the main phase transition (which directly corresponds to the single-step phase transition under acidic conditions) can be adjusted and predicted by the copolymer ratio of PNNPAM and PNIPMAM in the shell.

From an application-oriented point of view, the presented synthesis procedure for core-shell microgels seems to open a possibility for the development of a full set of temperature-dependent switchable microreactors by hybridization of the microgel particles with different metal nanoparticles. In addition, the particles could serve as membrane material with tunable permeation characteristics, by coating an inorganic membrane template with the different microgels [49].

## 4. Materials and Methods

### 4.1. Materials

If not indicated differently, all chemicals were purchased from Sigma Aldrich, Munich, Germany. *N*-isopropylacrylamide (NIPAM, TCI Germany GmbH, Eschborn, Germany, 97%) and *N*-isopropylmethacrylamide (NIPMAM, 97%) were recrystallized from *n*-hexane (VWR International, Darmstadt, Germany, p.a.). *N*,*N*′-methylenebisacrylamide (BIS, 99%), ammonium persulfate (APS, 99%), sodium dodecyl sulfate (SDS, 99%), acrylic acid (AAc, 99%) ethanol (HPLC grade, VWR International, Darmstadt, Germany), and polyethyleneimine (PEI, branched 99%) were used as obtained. Water was purified and deionized by an Arium pro VF system (Satorius Stedim Systems GmbH, Göttingen, Germany). *N-n*-propylacrylamide was synthesized as described by Hirano et al. [50] via a Schotten–Baumann reaction.

### 4.2. Core-Shell Microgel Synthesis

The core-shell microgels were synthesized via a two-step precipitation polymerization comparable to the procedure of Zeiser et al [36]. Both synthesis steps follow the typical synthesis route published by Pelton et al. [51]. For the synthesis of the PNIPAM–*co*–AAc core, NIPAM (11.55 mmol) and BIS (1.15 mmol, 10 mol %) were dissolved in 149 mL purified water. The mixture was purged with a constant nitrogen flux and heated up to 80 °C under constant stirring for one hour. Five minutes before the polymerization was initiated, SDS (0.18 mmol) and acrylic acid (0.58 mmol, 5 mol %) were added to the reaction mixture. The reaction was started by adding APS (0.41 mmol, 3.5 mol %) dissolved in 1 mL purified water. Afterwards, the reaction mixture was refluxed under constant stirring for four hours (80 °C). The core microgel was purified by five consecutive centrifugation (30,000 rpm, 45 min), decantation, and redispersion cycles with purified water. After the last centrifugation step, the microgel was redispersed in 80 mL purified, deionized water and a microgel suspension with a mass fraction of 1.66 wt% was yielded.

To generate core-shell microgels, a seeded precipitation polymerization was performed. Therefore, different molar ratios of NNPAM and NIPMAM (entire amount of substance 2.06 mmol), and BIS (0.16 mmol, 7.5 mol %) were added to a dispersion of the core-microgel (50 mL, 0.15 wt%) in purified water and equilibrated analogue to the core synthesis. The molar ratios of NNPAM/NIPMAM were 100/0 mol %, 75/25 mol %, 50/50 mol %, 25/75 mol %, and 0/100 mol %. Prior to the initiation with APS (0.14 mmol, 3.5 mol %), SDS was added to the reaction mixture. Analogous to the core synthesis, the core-shell microgel synthesis was refluxed for four hours and purified via centrifugation. After the last centrifugation step, the core-shell microgels were redispersed in 30 mL of purified water and stored at 4 °C. Samples of all core-shell microgels are available from Timo Brändel or Thomas Hellweg.

### 4.3. Atomic Force Microscopy

All measurements were performed on a FlexAFM (Nanosurf GmbH, Langen, Germany) in the dried state at room temperature in semi contact mode. The cantilever frequency was 100 kHz and the cantilevers (Tap300 AL-G, Budget Sensors, Innovative Solutions Bulgaria Ltd., Sofia, Bulgaria) had a spring constant of 40 N/m. For the sample preparation, a silicon wafer (Siegert Wafer GmbH, Aachen, Germany) was successively spin-coated (1000 rpm) with 0.1 mL of a PEI-solution (0.25 wt%) and a highly-diluted microgel suspension (c≤ 0.01 wt%). Prior to the PEI-coating, the wafer was cleaned with ethanol (HPLC grade) in a plasma cleaner (Zepto, Diener Electronics, Ebhausen, Germany). The resulting images were analyzed with GWYDDION [52]. To achieve good statistics for the height profiles, at least 32 particles were measured and averaged.

### 4.4. Photon Correlation Spectroscopy

For the sample preparation, all microgel suspensions were highly diluted ($c_{\mathrm{MG}}\leq 0.002\mathrm{wt}\%$) with buffer solutions (pH = 7 and pH = 4, 10 mM) in cylindrical cuvettes (Hellma GmbH & Co. KG, Müllheim, Germany).

The angle-dependent measurements were performed on a 3D-LS Spectrometer Pro (LS Instruments AG, Fribourg, Switzerland) equipped with a HeNe-Laser (JDSU 1145P, Thorlabs Inc., Newton, NJ, USA). The temperature was controlled via a thermostated index-matching decaline bath and adjusted to 10 and 55 °C. The equilibration time was at least 15 min for each temperature. Three measurements, with a measurement time of 2 min, were executed for each angle. The angles were adjusted from 35° to 120° with a step width of 5°. The resulting auto-correlation functions were analyzed with the method of cumulants [53]. The computed mean relaxation rates $\bar{\Gamma }$ were plotted against the square of the magnitude of the scattering vector $q^{2}$. From the linear dependency of $\bar{\Gamma }$ and $q^{2}$, the translational diffusion coefficient $D_{t}$ can be calculated (see Equation (3)).

(3) $$\bar{\Gamma }=D_{t}\;\cdot \;q^{2},$$

with

(4) $$q=\left|\vec{q}\right|=\frac{4\pi n}{\lambda }\sin\left(\frac{\theta }{2}\right).$$

Here, *n* is the refractive index of the sample, λ is the wavelength of the scattered light, and θ is the scattering angle. From the translational diffusion coefficient, the hydrodynamic radius $R_{h}$ can be subsequently calculated via the Stokes–Einstein equation, using the temperature *T*, the Boltzmann constant $k_{B}$, and the dynamic sample viscosity η.

(5) $$D_{t}=\frac{k_{B}T}{6\pi \eta R_{h}}.$$

For the temperature-dependent determination of the hydrodynamic radii, a fixed-angle PCS setup with a HeNe-Laser (HNL210L, Thorlabs Inc., Newton, NJ, USA) and an ALV-6010 multiple-τ-correlator (ALV GmbH, Langen, Germany) was used. The scattered light was collected by a single-mode fiber and forwarded to an ALV detection unit at an angle of 60°. The samples were tempered with a thermostated index-matching decaline bath. At each temperature, the equilibration time was 25 min, and five measurements with a measurement time of 5 min were performed. The data treatment was executed analogous to the angle-dependent PCS measurements.

### 4.5. Static Light Scattering

SLS measurements were performed on a 3D-LS Spectrometer Pro (see Photon Correlation Spectroscopy) on highly diluted samples at pH = 7. All samples were measured at 10 °C. To normalize the scattering intensity to absolute values, Equation (6) was used.

(6) $$I_{abs,\theta }=\frac{I_{s,\theta }-I_{sol,\theta }}{I_{tol,\theta }}\;\cdot \;R_{tol}\;\cdot \;{\left(\frac{n_{sol}}{n_{tol}}\right)}^{2}.$$

Here $I_{s,\theta }$ is the sample-scattering intensity at the angle θ, $I_{sol,\theta }$ is the respective solvent scattering intensity, and $n_{sol}$ is the refractive index of the solvent. Toluene was used as standard with the Rayleigh ratio $R_{tol}$, the refractive index $n_{tol}$, and the scattering intensity $I_{tol,\theta }$.

To obtain the radius of gyration $R_{g}$ of the microgel particles, a Guinier analysis of the scattering data was performed with a linear fit, according to Equation (7), up to a maximum value of $qR_{g}<1.3$.

(7) $$\ln\left(I(q)\right)=\ln\left(I\left(0\right)\right)-\frac{1}{3}R_{g}^{2}\;\cdot \;q^{2}.$$

## Figures and Tables

**Table 1:** Nominal shell monomer composition and sample names of all PNIPAM–*co*–AAc@PNNPAM–*co*–PNIPMAM core-shell microgels investigated in the present study.

Sample Name	PNNPAM Content/mol %	PNIPMAM Content/mol %
PNN100/PMAM0	100	0
PNN75/PMAM25	75	25
PNN50/PMAM50	50	50
PNN25/PMAM75	25	75
PNN0/PMAM100	0	100

**Table 2:** Lateral radii $R_{\mathrm{AFM}}$ of all dried core-shell microgel particles obtained from the AFM images presented in Figure 2. The errors were extracted from the standard deviations of $R_{\mathrm{AFM}}$. The lateral radius of the core microgels was 88 nm (No corrections with respect to the tip profile were applied).

Sample Name	$R_{\mathrm{AFM}}$/nm
PNN100/PMAM0	108±32
PNN75/PMAM25	150±25
PNN50/PMAM50	143±25
PNN25/PMAM75	135±23
PNN0/PMAM100	158±24

**Table 3:** Polydispersity index (PDI)-values of all core-shell microgels in the swollen state, obtained from the cumulant analysis of the angle-dependent PCS measurements at 10 °C.

Sample Name	PDI (pH 4)/%	PDI (pH 7)/%	Average PDI/%
PNN100/PMAM0	4.3	3.4	3.9
PNN75/PMAM25	4.3	4.0	4.2
PNN50/PMAM50	4.8	4.4	4.6
PNN25/PMAM75	4.2	3.7	4.0
PNN0/PMAM100	4.4	2.8	3.7

**Figure A1:**
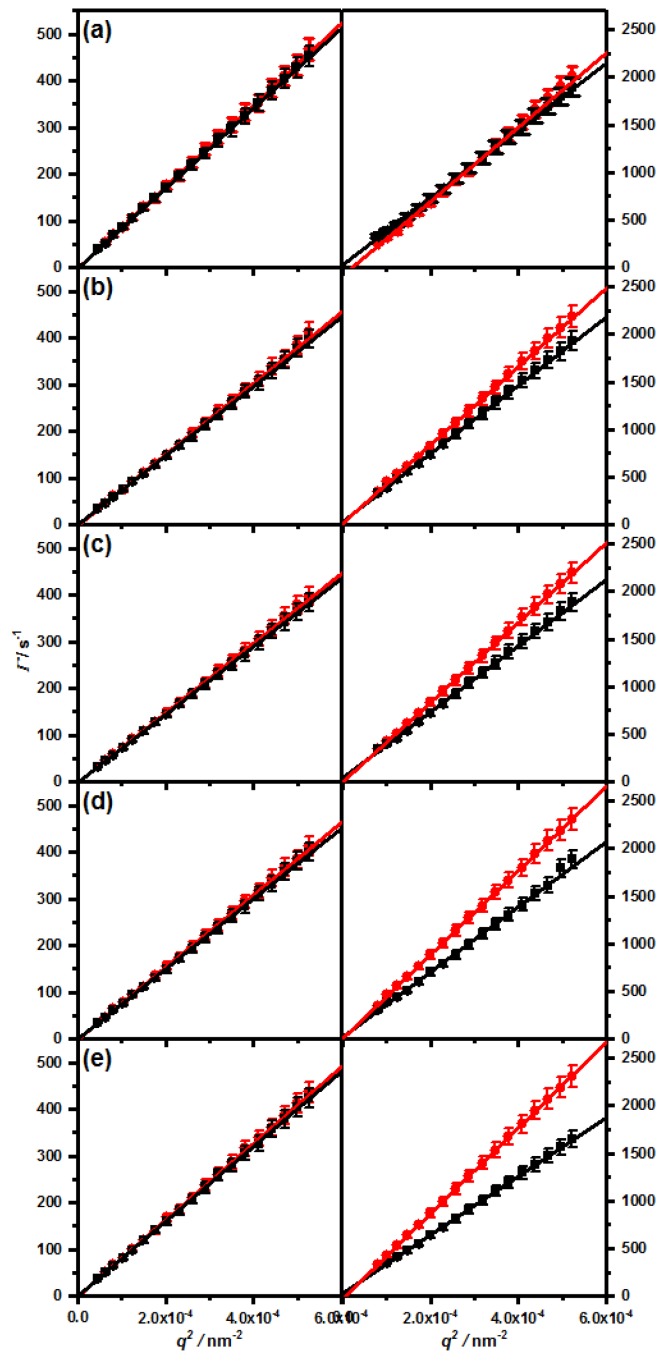
Angle-dependent PCS measurements of (**a**) PNN100/PMAM0; (**b**) PNN75/PMAM25; (**c**) PNN50/PMAM50; (**d**) PNN25/PMAM75 and (**e**) PNN0/PMAM100 at pH7 (black squares) and pH4 (red circles). All measurements were performed at 10 (left part of figure) and 55 °C (right part of figure).

## Appendix A

In Figure A2, clear evidence for the core-shell architecture can be deduced from two facts. Firstly, the size of the microgels in the swollen state increases from the core microgels to the core-shell microgels. According to our findings in the results section, the increase in size is inferior for the sample PNN100/PMAM0 compared to the sample PNN0/PMAM100. Secondly, the course of the swelling curves is significantly different for both core-shell samples compared to the core microgels.

## References

[1] Karg M., Pich A., Hellweg T., Hoare T., Lyon L.A., Crassous J.J., Suzuki D., Gumerov R.A., Schneider S., Potemkin I.I. (2019). Nanogels and Microgels: From Model Colloids to Applications, Recent Developments, and Future Trends. Langmuir.

[2] Plamper F.A., Richtering W. (2017). Functional Microgels and Microgel Systems. Acc. Chem. Res..

[3] Hellweg T. (2013). Responsive core-shell microgels: Synthesis, characterization, and possible applications. J. Polym. Sci. Part B Polym. Phys..

[4] Gan D., Lyon L.A. (2001). Tunable Swelling Kinetics in Core–Shell Hydrogel Nanoparticles. J. Am. Chem. Soc..

[5] Richtering W., Pich A. (2012). The special behaviors of responsive core–shell nanogels. Soft Matter.

[6] Lee S.M., Bae Y.C. (2014). Swelling Behaviors of Doubly Thermosensitive Core–Shell Nanoparticle Gels. Macromolecules.

[7] Cors M., Wrede O., Genix A.C., Anselmetti D., Oberdisse J., Hellweg T. (2017). Core-Shell Microgel-Based Surface Coatings with Linear Thermoresponse. Langmuir.

[8] Dimitrov I., Trzebicka B., Müller A.H., Dworak A., Tsvetanov C.B. (2007). Thermosensitive water-soluble copolymers with doubly responsive reversibly interacting entities. Progr. Polym. Sci..

[9] Lu Y., Ballauff M. (2011). Thermosensitive core–shell microgels: From colloidal model systems to nanoreactors. Progr. Polym. Sci..

[10] Balaceanu A., Verkh Y., Demco D.E., Möller M., Pich A. (2013). Correlated Morphological Changes in the Volume Temperature Transition of Core–Shell Microgels. Macromolecules.

[11] Karg M., Wellert S., Prevost S., Schweins R., Dewhurst C., Liz-Marzán L.M., Hellweg T. (2011). Well defined hybrid PNIPAM core-shell microgels: Size variation of the silica nanoparticle core. Colloid Polym. Sci..

[12] Dulle M., Jaber S., Rosenfeldt S., Radulescu A., Förster S., Mulvaney P., Karg M. (2015). Plasmonic gold–poly(N-isopropylacrylamide) core–shell colloids with homogeneous density profiles: A small angle scattering study. Phys. Chem. Chem. Phys..

[13] Silan C., Akcali A., Otkun M.T., Ozbey N., Butun S., Ozay O., Sahiner N. (2012). Novel hydrogel particles and their IPN films as drug delivery systems with antibacterial properties. Colloids Surf. B Biointerfaces.

[14] Rudyak V.Y., Gavrilov A.A., Kozhunova E.Y., Chertovich A.V. (2018). Shell-corona microgels from double interpenetrating networks. Soft Matter.

[15] Schmid A.J., Dubbert J., Rudov A.A., Pedersen J.S., Lindner P., Karg M., Potemkin I.I., Richtering W. (2016). Multi-Shell Hollow Nanogels with Responsive Shell Permeability. Sci. Rep..

[16] Brugnoni M., Scotti A., Rudov A.A., Gelissen A.P.H., Caumanns T., Radulescu A., Eckert T., Pich A., Potemkin I.I., Richtering W. (2018). Swelling of a Responsive Network within Different Constraints in Multi-Thermosensitive Microgels. Macromolecules.

[17] Leite D.C., Kakorin S., Hertle Y., Hellweg T., da Silveira N.P. (2018). Smart Starch-Poly(N-isopropylacrylamide) Hybrid Microgels: Synthesis, Structure, and Swelling Behavior. Langmuir.

[18] Schachschal S., Balaceanu A., Melian C., Demco D.E., Eckert T., Richtering W., Pich A. (2010). Polyampholyte Microgels with Anionic Core and Cationic Shell. Macromolecules.

[19] Gelissen A.P.H., Scotti A., Turnhoff S.K., Janssen C., Radulescu A., Pich A., Rudov A.A., Potemkin I.I., Richtering W. (2018). An anionic shell shields a cationic core allowing for uptake and release of polyelectrolytes within core-shell responsive microgels. Soft Matter.

[20] Kratz K., Eimer W. (1998). Swelling properties of colloidal poly(N-Isopropylacrylamide) microgels in solution. Berichte der Bunsengesellschaft für Physikalische Chemie.

[21] Pelton R. (2000). Temperature-sensitive aqueous microgels. Adv. Colloid Interface Sci..

[22] Das M., Zhang H., Kumacheva E. (2006). Microgels: Old Materials with New Applications. Annu. Rev. Mater. Res..

[23] Pich A.Z., Adler H.J.P. (2007). Composite aqueous microgels: An overview of recent advances in synthesis, characterization and application. Polym. Int..

[24] Snowden M.J., Chowdhry B.Z., Vincent B., Morris G.E. (1996). Colloidal copolymer microgels of N-isopropylacrylamide and acrylic acid: pH, ionic strength and temperature effects. J. Chem. Soc. Faraday Trans..

[25] Hoare T., Pelton R. (2006). Titrametric characterization of pH-induced phase transitions in functionalized microgels. Langmuir.

[26] Wang Z., Pelton R. (2014). Aminated thermoresponsive microgels prepared from the Hofmann rearrangement of amides without side reactions. Langmuir.

[27] Scherzinger C., Balaceanu A., Hofmann C.H., Schwarz A., Leonhard K., Pich A., Richtering W. (2015). Cononsolvency of mono- and di-alkyl N-substituted poly(acrylamide)s and poly(vinyl caprolactam). Polymer.

[28] Wedel B., Hertle Y., Wrede O., Bookhold J., Hellweg T. (2016). Smart Homopolymer Microgels: Influence of the Monomer Structure on the Particle Properties. Polymers.

[29] Kodlekere P., Andrew Lyon L. (2018). Microgel core/shell architectures as targeted agents for fibrinolysis. Biomater. Sci..

[30] Wu C., Zhou S., Au-yeung S.C.F., Jiang S. (1996). Volume phase transition of spherical microgel particles. Angewandte Makromolekulare Chemie.

[31] Wedel B., Zeiser M., Hellweg T. (2012). Non NIPAM Based Smart Microgels: Systematic Variation of the Volume Phase Transition Temperature by Copolymerization. Zeitschrift für Physikalische Chemie.

[32] Keerl M., Pedersen J.S., Richtering W. (2009). Temperature sensitive copolymer microgels with nanophase separated structure. J. Am. Chem. Soc..

[33] Wiehemeier L., Cors M., Wrede O., Oberdisse J., Hellweg T., Kottke T. (2019). Swelling behavior of core-shell microgels in H_2_O, analysed by temperature-dependent FTIR spectroscopy. Phys. Chem. Chem. Phys..

[34] Jones C.D., Lyon L.A. (2000). Synthesis and Characterization of Multiresponsive Core-Shell Microgels. Macromolecules.

[35] Berndt I., Pedersen J.S., Richtering W. (2006). Temperature-sensitive core-shell microgel particles with dense shell. Angew. Chem. Int. Ed..

[36] Zeiser M., Freudensprung I., Hellweg T. (2012). Linearly thermoresponsive core–shell microgels: Towards a new class of nanoactuators. Polymer.

[37] Berndt I., Pedersen J.S., Lindner P., Richtering W. (2006). Influence of shell thickness and cross-link density on the structure of temperature-sensitive poly-N-isopropylacrylamide-poly-N-isopropylmethacrylamide core-shell microgels investigated by small-angle neutron scattering. Langmuir.

[38] Lapeyre V., Renaudie N., Dechezelles J.F., Saadaoui H., Ravaine S., Ravaine V. (2009). Multiresponsive Hybrid Microgels and Hollow Capsules with a Layered Structure. Langmuir.

[39] Brändel T., Sabadasch V., Hannappel Y., Hellweg T. (2019). Improved Smart Microgel Carriers for Catalytic Silver Nanoparticles. ACS Omega.

[40] Suzuki D., Kawaguchi H. (2005). Gold nanoparticle localization at the core surface by using thermosensitive core-shell particles as a template. Langmuir.

[41] Kureha T., Nagase Y., Suzuki D. (2018). High Reusability of Catalytically Active Gold Nanoparticles Immobilized in Core–Shell Hydrogel Microspheres. ACS Omega.

[42] Brändel T., Wiehemeier L., Kottke T., Hellweg T. (2017). Microphase separation of smart double-responsive copolymer microgels studied by local fluorescence probes. Polymer.

[43] Rueden C.T., Schindelin J., Hiner M.C., DeZonia B.E., Walter A.E., Arena E.T., Eliceiri K.W. (2017). ImageJ2: ImageJ for the next generation of scientific image data. BMC Bioinform..

[44] Burmistrova A., Richter M., Eisele M., Üzüm C., von Klitzing R. (2011). The Effect of Co-Monomer Content on the Swelling/Shrinking and Mechanical behavior of Individually Adsorbed PNIPAM Microgel Particles. Polymers.

[45] Hyatt J.S., Do C., Hu X., Choi H.S., Kim J.W., Lyon L.A., Fernandez-Nieves A. (2015). Segregation of mass at the periphery of N -isopropylacrylamide-co-acrylic-acid microgels at high temperatures. Phys. Rev. E.

[46] Cors M., Wiehemeier L., Hertle Y., Feoktystov A., Cousin F., Hellweg T., Oberdisse J. (2018). Determination of Internal Density Profiles of Smart Acrylamide-Based Microgels by Small-Angle Neutron Scattering: A Multishell Reverse Monte Carlo Approach. Langmuir.

[47] Gelissen A.P.H., Oppermann A., Caumanns T., Hebbeker P., Turnhoff S.K., Tiwari R., Eisold S., Simon U., Lu Y., Mayer J. (2016). 3D Structures of Responsive Nanocompartmentalized Microgels. Nano Lett..

[48] Bergmann S., Wrede O., Huser T., Hellweg T. (2018). Super-resolution optical microscopy resolves network morphology of smart colloidal microgels. Phys. Chem. Chem. Phys..

[49] Barth M., Wiese M., Ogieglo W., Go D., Kuehne A., Wessling M. (2018). Monolayer microgel composite membranes with tunable permeability. J. Membr. Sci..

[50] Hirano T., Nakamura K., Kamikubo T., Ishii S., Tani K., Mori T., Sato T. (2008). Hydrogen-bond-assisted syndiotactic-specific radical polymerizations ofN-alkylacrylamides: The effect of theN-substituents on the stereospecificities and unusual large hysteresis in the phase-transition behavior of aqueous solution of syndiotactic poly(N-n-propylacrylamide). J. Polym. Sci. Part A Polym. Chem..

[51] Pelton R.H., Chibante P. (1986). Preparation of aqueous latices with N-isopropylacrylamide. Colloids Surf..

[52] Nečas D., Klapetek P. (2012). Gwyddion: An open-source software for SPM data analysis. Open Phys..

[53] Koppel D.E. (1972). Analysis of Macromolecular Polydispersity in Intensity Correlation Spectroscopy: The Method of Cumulants. J. Chem. Phys..

